# How do Gambling Providers Use the Social Network Twitter in Germany? An Explorative Mixed-Methods Topic Modeling Approach

**DOI:** 10.1007/s10899-022-10158-y

**Published:** 2022-09-14

**Authors:** Johannes Singer, Vadim Kufenko, Andrea Wöhr, Marius Wuketich, Steffen Otterbach

**Affiliations:** 1 Gambling Research Center, University of Hohenheim, Forschungsstelle Glücksspiel (502), Schwerzstraße 44, 70599 Stuttgart, Germany; 2 Institute for Health Care & Public Management, University of Hohenheim, 70599 Stuttgart, Germany; 3 Institute of Labor Economics (IZA), Bonn, Germany

**Keywords:** Advertising, Gambling, Marketing, Social media, Semi-supervised topic modeling, Twitter

## Abstract

This study examines the social media activities of gambling providers in Germany, focusing on the platform Twitter. A collection of 34.151 tweets from 13 Twitter accounts was made, representing casinos, sports betting, state lotteries, social lotteries and lottery brokers. We apply an explorative mixed-methods approach, integrating a summative content analysis together with a semi-supervised guided topic modeling approach, to analyse frequency, number of followers, interaction and content of Twitter messages, and work out differences among the individual providers. The results show that Twitter does not seem to be particularly important for gambling providers who are active in Germany. Regarding outreach, frequency of tweets and interaction, Twitter plays a much smaller role than, for example, in the UK. The potential for Twitter to be an advertising, marketing and interaction channel has not yet been fully exploited, which would make it a perfect moment for developing an appropriate regulatory framework. Overall, the results of semi-supervised topic modeling show that providers most often use informative content (*news*) and combine it with other, usually less neutral content. It is alarming that many providers make little or no use of *Responsible Gambling* messages. Even though the activities are presently on a low level, they contribute to the normalization of gambling, setting incentives for increasing gambling participation. Children and youth form a large segment of Twitter users. Potential harm can arise especially for this group because of the lack of enforcement of any age limit.

## Introduction

The activities of gambling providers in the social media, to our knowledge, have only been researched in Australia and the UK (Bradley & James, 2019; Gainsbury et al., 2016a; Houghton et al., 2019; Killick & Griffiths, 2020). However, the question regarding how social media are used as advertising platforms is also relevant for Germany, particularly as the new State Treaty on Gambling (GlüStV, 2020, 2021), entered into force on the first of July 2021 legalizes forms of online gambling that have been prohibited or, during the last months before the State Treaty came into force, tolerated. Providers of online casinos, online poker, virtual slot machine games and sports betting will be permitted from July 2021 onwards (Koch, 2021), assuming that they hold a corresponding license. The social media activities of gambling providers in a semi-legal environment, to our knowledge, have not yet been examined.

Legalization of online gambling is seen critically by addiction experts, as the internet offers an almost endless number of opportunities for gambling and facilitates access to it (Griffiths & Barnes, 2008). Gambling providers on the other hand welcome the new opportunities. Many providers already use social media to market and advertise their brands and products, maintaining several accounts on different social networks in order to reach as many users as possible (Gainsbury et al., 2016a). Not only can they advertise their products in a global network at low cost and get in direct contact with current and potential future customers (Parke et al., 2014), they also use social media to build brand awareness (Barreda et al., 2015).

Despite this situation, research on social media advertising for gambling is scarce (Torrance et al., 2021), and, to our knowledge, non-existing for Germany. This research gap may be partly related to methodological difficulties in quantifying the influence of such advertising strategies on gambling behavior (Binde, 2014). Barely any attempts of a quantitative analysis using unsupervised and semi-supervised machine learning methods have been made in order to analyse the advertisement activities of the German gambling providers. However, no one will deny that marketing and advertising on social media are gaining importance and must, therefore, be duly investigated.

Over the past decade, the use of social media has grown and this trend is expected to continue (Duggan et al., 2016). New offers and applications such as TikTok or Clubhouse frequently appear online. This growth also applies to the “big players”, as can be seen from the example of Twitter. The social network recorded a 20% increase in daily monetizable users worldwide compared to the previous year, reaching 199 million in Q1 2021 (Twitter, 2021). In Germany, 31% of the population uses social media daily; among 14- to 29-year-olds, the rate is as high as 66%. Alongside Facebook and Instagram, Twitter is used by 2% of the population; among those under the age of 30, also 2% use the microblogging social media platform daily (Beisch & Koch, 2021).

Although Twitter enjoys great popularity among gambling operators (Gainsbury et al., 2015), the advertising strategies can be seen as problematic in that, for example, warnings and notices are very rarely included (Bradley & James, 2019; Gainsbury et al., 2016b; Houghton et al., 2019; Killick & Griffiths, 2020; Sproston et al., 2015; Thomas et al., 2015). With the help of positive framing, gambling is portrayed as a social and leisure activity like any other (Binde, 2014; Sproston et al., 2015; Gainsbury et al., 2016a, b; Bradley & James, 2019; Ginnis, 2019). Different types of sports, especially football, are inextricably connected to gambling offers (Cassidy & Ovenden, 2017; Houghton et al., 2019; Lopez-Gonzalez & Griffiths, 2018). Vulnerable individuals, particularly young people, are exposed to enormous amounts of advertising, especially since registration on Twitter requires a minimum age of only 13 (Twitter, 2020), and this restriction is rarely checked. This is an alarming situation since young people are more likely to be influenced by gambling advertisements (Hanss et al., 2015). Besides, advertising aims to increase the number of gamblers and may therefore increase gambling-related harm (Parke et al., 2014). People with gambling disorder are likely to intensify their gambling behavior (Hing et al., 2014, 2015), gambling more often and more riskily (Hing et al., 2018).

From a researcher’s point of view, investigating the communication via Twitter is attractive because of the breadth and depth of the data on the exchange between gambling providers and their target audiences, available for quantitative analysis. The well-established Twitter Application Programming Interface (API) allows to extract Twitter-objects with rich metadata, which can be used for further analysis. Although analysing short text corpora can be challenging, a number of works has recently emerged (Berger et al., 2020; Liu et al., 2017; Steuber et al., 2022) paving the way to further studies on text analysis of Twitter accounts of different firms.

The growing complexity and availability of gambling (Winters et al., 2019; Lawn et al., 2020) is reflected in an increase in the prevalence, diversity and intensity of advertising (Browne et al., 2019; Newall, 2017). Thus, contemporary gambling marketing represents a multi-layered mix of mass media promotion, consumer marketing and subtle marketing, in which advertising is a key component (Newall et al., 2019). Given this fact and the multitude of possible concerns, it is almost negligent that policy makers have paid little attention to social media advertising, for example, by suggesting guidelines or forms of legal regulation.

In the following study, the advertising practices of various gambling providers on Twitter will be examined to get a picture of the situation in Germany. Since there is no freely accessible information on paid advertising, the analysis is limited purely to the activity of the corresponding accounts. First, the frequency of the tweets and interactions with the tweets, i.e., likes and retweets, are described. Second, the contents of the tweets are analysed. Strategies and mechanisms used, such as URLs, hashtags and replies, are considered. Based on a summative content analysis of a random sample, a topic modeling approach is applied to determine the distribution of topics for the complete corpus. The limitations of previous research approaches are taken into account and the novelty of our work is related to i) the choice of the mixed-methods approach, thus applying both qualitative and quantitative methods of empirical social research; ii) analysis of the situation in Germany and iii) consideration of providers from different sectors (Torrance et al., 2021).

## Data and Methodology

### Identification of Twitter Accounts

Based on the report from the gambling supervisory authorities of the federal states of Germany (Hessisches Ministerium des Inneren und für Sport, 2020), various sectors and providers were identified: casinos, slot machines, social lotteries, sports betting, state lotteries, and lottery brokers, resulting in a total of 126 possible gambling providers. Following the recommendations given in Bradley and James (2019), only Twitter accounts were selected that met the following criteria: (1) the Twitter account had to be in German; (2) there had to be a certain level of activity (i.e., at least one tweet per week) and (3) the two Twitter accounts with the largest number of followers per sector were included.

In the case of sports betting providers, an exception from the third criterion was made in that accounts from six providers were chosen (instead of two). Thus, a broader data base was obtained, making the results more readily comparable with previous research, which primarily focuses on sports betting providers in Australia and the United Kingdom (Bradley & James, 2019; Gainsbury et al., 2015; Houghton et al., 2019; Killick & Griffiths, 2020).

The situation with the Twitter accounts of casinos and slot machine providers was different: these do not tend to actively use Twitter and some accounts were inactive or had not been updated for a long time. Therefore, only one account from the casino sector and none from the slot machine sector met the criteria. In total, 13 accounts were included in the study.

### Sample

Table 1 gives an overview of the data collected: 34,151 tweets from 13 accounts were collected on April 8, 2021. Account holders were the sports betting providers *ADMIRALBET, bet-at-home, bwin Sportwetten, mybet, Tipico and Unibet Sportwetten*;^1^ the state lotteries *Lotto BW* and *LOTTO Bayern*; the social lotteries *Aktion Mensch e.V.* and *Sportlotterie*; the lottery brokers *LOTTO24.de* and *Lottoland.com* and the casino provider *Spielbanken Bayern*.^2^ The gambling providers generated 32,861 tweets (96.22%); 1,290 tweets (3.78%) were retweets. The average number of shared tweets varied from 15.19 (*bwin Sportwetten*) to 0.42 (*Spielbanken Bayern*).

**Table 1:** Twitter account information by provider

Gambling provider	Account starting date	Followers	Number of tweets		
			Total since start	Per day	Collected via API
*State lotteries*					
Lotto BW	2014–09-22	1735	8781	3.67	3200
LOTTO Bayern	2015–06-11	1452	3907	1.84	3198
*Social lotteries*					
Aktion Mensch e.V	2009–06-29	78,105	31,254	7.27	3199
Sportlotterie	2014–02-03	1149	1911	0.73	1909
*Lottery brokers*					
Lottoland.com	2012–02-02	1225	3283	0.98	3198
LOTTO24.de	2012–11-14	2478	5804	1.89	3199
*Casino*					
Spielbanken Bayern	2013–01-02	132	1258	0.42	1258
*Sports betting*					
ADMIRALBET	2010–01-19	1713	4851	1.18	3200
bet-at-home	2011–08-01	1813	11,314	3.20	3198
bwin Sportwetten	2013–05-23	2938	43,692	15.19	3200
mybet	2019–03-27	20	434	0.58	434
Tipico	2016–04-13	3799	2493	1.37	2488
Unibet Sportwetten	2018–02-15	4521	2497	2.18	2470

The provider whose account registration dated back the longest was the social lottery *Aktion Mensch e.V.*, whereas the sports betting provider *mybet* held the most recent account. Since registration, *bwin Sportwetten* had posted the largest number of tweets (43,692) and *mybet* the fewest (434). *Aktion Mensch e.V.* had the highest number of followers with 78,105, *mybet* the fewest (20).

The observation period starts with the registration of the most recent account (03/27/2019) and ends about two years later (04/08/2021). Thus, a consistent time period in which all accounts were active is ensured, allowing for a meaningful comparison of frequency, interaction and content.

### Methods

In this study, we use an exploratory mixed methods approach, where an initial qualitative phase of data collection an analysis is followed by a phase of quantitative data collection and analysis. Thus, we address the limitations of previous studies that rely on a single method (Bradley & James, 2019; Houghton et al., 2019; Killick & Griffiths, 2020). The methods can be categorized into the ones related to data acquisition, qualitative analysis, preprocessing, quantitative analysis and evaluation.

In the first part of our analysis dedicated to data aquisition, we use the statistics program R and the package rtweet (Kearney, 2019) to collect the most recent tweets within the observation period for each provider. A maximum of 3200 tweets (excluding retweets) could be collected per account. This requires a Twitter developer account and a personalized access key to the Twitter API. This data gives insight into the frequency of tweets, number of followers and interaction with the tweets. In all, 18,051 tweets were collected for the observation period.

In the second part of our analysis, we carry out a qualitative summative content analysis (Hsieh & Shannon, 2005) to examine the content of the tweets. In a first step, we randomly select 10 tweets per provider, resulting in 130 tweets, which serve as a basis for the development of a coding scheme. Using an inductive approach, we assign the content of the tweets to specific codes. Code identification provides a way to capture content, detect and classify messages into interpretable topics. This qualitative classification is based on the domain knowledge about the gambling market and products specific to Germany. The results of this qualitative classification are valuable per se, yet they relate to the selected subsample of tweets. In order to test whether the given distribution of topics can be generalized for the complete sample of tweets, a semi-supervised guided topic modeling approach is applied. The results of the qualitative analysis are used to initialize the topics and guide the algorithm.

Next, we summarize and condense the codes. Eight categories can be identified, some of which have already been described in previous studies (Houghton et al., 2019; Killick & Griffiths, 2020; Thomas et al., 2015), namely, *additional information*, *interaction*, *marketing*, *news*, *product advertising*, *Responsible Gambling*, *results*, and *other*. These categories are then applied to a random sample of 50 tweets per provider, resulting in 650 tweets. The number of 50 was chosen due to the difference in activity between the providers, with *Aktion Mensch e.V.* posting 3.194 tweets and *Sportlotterie* only 66 tweets during the observation period. The above-mentioned categories and the related words are used as seeds, or guides, for the semi-supervised topic modeling.

For the quantitative analysis we use the Python programming language in order to preprocess the tweets,^3^ assemble the corpus and conduct semi-supervised topic modeling. This allows us to apply the findings of the summative content analysis to all 18,051 tweets of the observation period and evaluate the generalization of our analysis. A diligent preprocessing of tweets was necessary in order to proceed to the quantitative analysis. The preprocessing routines involved global ones,^4^ including but not limited to capitalization of text to lowercase, converting German diacritics into their non-diacritic character combinations, removal of German stop words and extraction of text from images,^5^ actively used by the providers to visualize their messages. Lemmatization of the words was carried out using a special tagger,^6^ tailored for German language (Wartena, 2019). Since usually preprocessing involves removal of special characters and numbers, local or provider-specific pre-processing involved translating certain frequently observed objects, like the hotline telephone numbers for prevention, certain symbols and icons or specific product names containing numbers, into interpretable words.

Regarding the semi-supervised topic modeling a number of methods has been considered. As Steuber et al. (2022) demonstrated, guided or seeded Latent Dirichlet Allocation (LDA as in Blei et al., 2003) can be successfully applied to Twitter data. However, Gallagher et al. (2017) noted that LDA is based on a number of generative assumptions on the distribution of topics over words, which are often unrealistic and result in rather narrow topic definitions. Therefore, we have decided to apply the Correlation Explanation (CorEx) topic model, treating topics as latent factors. The CorEx model is relatively new and to our knowledge this is a first time that this model is used on German Twitter data. There are different ways of guiding the algorithms. In Steuber et al. (2022) the topic distribution and related words were identified using clustering analysis, which yielded rather dispersed results for some topics. Although such approaches can be fruitful, our mixed methods strategy has a decisive advantage: we use the qualitative analysis to feed the extensive domain knowledge into the seeds of the topic modeling algorithm.

The codes from the summative content analysis act as a guide for the CorEx model. The specification of anchor words enables the topic modeling procedure to assign certain keywords to the different content categories. To evaluate whether the initial topic distribution and classification of tweets from the qualitative analysis can be generalized to a larger corpus, we use an inter-rater reliability and agreement approach resembling the one used in Goh et al. (2020): For the qualitative coding of the 650 randomly selected tweets we compare the corresponding results from the CorEx classification using Fleiss’ (1971) κ from the pyirr package (Rick de Klerk, 2022).

## Results

### Descriptive Analysis

The first steps in our analysis are of a descriptive nature and represent frequencies and descriptive statistics on the Twitter messages for a general overview. Below we summarize the main descriptive findings from the tables, which can be found in the Appendix.

#### Frequency of the Tweets

The activity of the providers varied considerably (see “Appendix, Table 7”). *bwin Sportwetten* (3197) and *Aktion Mensch* e.V. (3191) posted the most tweets in the observation period, *Sportlotterie* the fewest (66). The highest number of tweets per day came from *bwin Sportwetten* (4.30) and *Aktion Mensch e.V.* (4.29), the fewest from *Sportlotterie* (0.09).

#### Interaction with the Tweets

Actions such as retweeting, commenting or liking a tweet, and the use of hashtags create additional content and visibility to a wider audience. Consequently, both number of followers and level of interaction are assessed to evaluate the activities of the individual providers. The number of retweets and likes is shown in Table 8 (see “Appendix”).

It is of note that gambling providers sometimes retweet the content of other accounts. This is especially common among sports betting providers, who, for example, often retweet the messages of major football clubs such as FC Bayern Munich. These retweets (*n* = 694) are excluded from the analysis so that we can exclusively examine the content created by the gambling providers themselves.

#### Content of the Tweets

Finally, we examine the number of images and URLs included, as well as the providers’ responses to user comments (see “Appendix, Table 9”). Images were used in varying frequencies. Whereas *Spielbanken Bayern* used images in almost every tweet (94.04%), *ADMIRALBET* almost never did (4.00%). Most providers added one or several URLs to their tweets, linking their websites or other content. Even *LOTTO24.de*, the provider with the lowest number of URLs, used a URL in one third of its tweets (33.97%). In contrast, replies to tweets were rare, with the exception of *LOTTO24.de,* who responded to user questions or comments in 40.38% of all cases.

Next, we look at hashtags. Hashtags enable providers to relate their brand and products to certain topics. Frequent hashtags are displayed on the start screen of the users, increasing visibility. Table 10 (see “Appendix”) presents the most frequent hashtags. First was *lotto6aus49* (number lottery), followed by *bundesliga* (German term for *national league*) and *jackpot*.

Table 11 (see “Appendix”) shows the top 5 hashtags for each provider. Providers from the same sector mostly used identical or similar hashtags. Sports betting providers, for example, addressed sports teams, sporting events, (betting) odds and facts about sports events. The social lottery *Aktion Mensch e.V.* addressed primarily social issues, with hashtags such as *inclusion*, *disability*, *accessibility* and *participation*. In contrast, the social lottery *Sportlotterie* exclusively referred to their own company and brand. Hashtags could clearly be assigned to certain sectors; the only intersection appeared between lotteries and lottery brokers, whose product interests are closely related: the state lotteries *Lotto BW* and *LOTTO Bayern* and the lottery brokers *Lottoland.com* and *LOTTO24.de* referred to their products (e.g., *lottozahlen* and *lotto6aus49*) and advertised high chances of winning (e.g., *jackpot* and *eurojackpot*).

The majority of the hashtags are related to the contents. Only few hashtags describe characteristics or qualities of the provider using them. Several sports betting providers stress the high quality of their offer (*quotenboost*, *topquoten*, *bonus*), sometimes in combination with their own brand name (*tipicotopfakt*, *mybetmeister*). The state lottery *LOTTO Bayern* emphasizes its legal status (*legalbeimoriginal*) and proximity (*bayern*). The hashtag *glücklichmacher* used by the lottery broker *LOTTO24.de,* suggests that by using the company’s products, the users will be made “happier”.

### Qualitative and Quantitative Content Analysis

In the first step, 50 randomly selected tweets per provider are categorized to develop a coding scheme based on associated codes and keywords for the entire dataset. After the 650 tweets were coded by the first researcher, a second researcher applied the categories to 40% of the data. We calculate Cohen's (1960) κ as a measure of inter-rater reliability. Table 2 shows consistently high inter-rater reliability with κ values larger 0.84 for seven out of eight categories. On average the Cohen's κ was around 0.77 with only one outlier.^7^

**Table 2:** Content categories of the 650 randomly selected tweets (50 per provider)

Category	Qualitative analysis (*n*^a^ = 1115)			CorEx (*n* = 793)	
	*n*	%	Cohen’s κ^b^	*n*	%
1. Product advertising	165	15	0.9300	167	21
2. Additional information	159	14	0.9385	157	20
3. Results	89	8	0.8710	91	11
4. Marketing	190	17	0.8438	148	19
5. Interaction	15	1	0.4611	17	2
6. Responsible Gambling	98	9	0.9593	54	7
7. News	350	31	0.8924	123	16
8. Other	49	4	0.9168	36	5

The analysis is based on 650 randomly selected tweets (50 tweets per provider) during the observation period from 2019–03–27 to 2021–04–08

^a^ *n* means the number of identified content categories for all 13 providers. The number of content categories is higher than the number of tweets, as a tweet can be classified in several categories

^b^ The values for Cohen's κ refer to the agreement of the two researchers regarding the qualitative coding of 650 randomly selected tweets. The comparison of the agreement between the qualitative content analysis and the semi-supervised topic modeling regarding the 650 randomly selected tweets is shown in Table 12 (see “Appendix”)

The eight identified topics and the associated codes and keywords from the summative content analysis serve as a guide for the semi-supervised CorEx topic model (Gallagher et al., 2017). It is important to note that associated codes and keywords are identified on a provider-specific basis, as providers operate in different gambling sectors and therefore use different jargon, house style and wording in their Twitter messages. Also with regard to the eight categories, it should be mentioned that (i) not every provider takes up all eight categories and (ii) individual messages can be assigned to more than one category. By specifying provider-specific anchor words, the topic modeling procedure is able to capture the specific characteristics of each provider as accurately as possible and optimize pre-processing for the entire dataset.

Given the provider-specific anchor words, we perform the CorEx topic modeling for each provider and compare the match with the qualitative summative content analysis. Table 2 shows the level of correspondence for the 650 randomly selected and previously qualitatively classified Twitter messages. With the exception of the categories *news* and *Responsible Gambling*, a relatively high level of matching can be seen. While the qualitative content analysis assigns 165 of the 650 messages to the category *product advertising*, the CorEx topic modeling assigns 167. A similar result can be seen in the categories *additional information* (159 vs. 157) and *results* (89 vs. 91). It is noteworthy that in the qualitative categorization tweets are much more frequently assigned to more than one category, i.e. 650 tweets are assigned to a total of 1,115 categories, while in the CorEx topic modeling it is 793. The relatively poor match for the *Responsible Gambling* category can be explained by the fact that images and symbols are often used for this. *Bet-at-home*, for example, uses only an emoticon-sized symbol to indicate the age restriction. *Sportlotterie* also often uses images to indicate *Responsible Gambling*.

In the next step, we further examine the correspondence of the qualitative and quantitative topic analysis by calculating Fleiss κ for each provider and category (see Table 12). Given that not every provider´s Twitter messages cover all eight categories, 67 categories are identified for comparison.^8^ The inter-rater reliability shows substantial agreement on 58 categories (87%) having a positive κ, and 50 categories (75%) additionally having a *p*-value below 5%, indicating that the agreement between the qualitative and the quantitative analysis is significantly different from a chance agreement (Table 3). CorEx yielded relatively high coherence values,^9^ calculated as in Syed and Spruit (2017), between 0.45 and 0.63 (see “Appendix”, Table 13).

**Table 3:** Inter-rater reliability of the 67^a^ content categories of all 13 providers based on the 650 randomly selected tweets (50 per provider)

Value	*n*	%
κ ( +)	58	87
p-value (< 0.05)	55	82
κ ( +) & *p*-value (< 0.05)	50	75
κ ( −) & *p*-value (< 0.05)	5	7

^a ^ In total, 67 content categories can be identified for all 13 gambling providers. The provider whose content can be classified into the fewest categories (3) is Aktion Mensch e.V. The largest number of content categories (7) can be found at several providers (e.g. Lotto BW)

Finally, we apply the semi-supervised CorEx topic model to the entire dataset containing 18,051 tweets. Again, a single tweet can be assigned to one or more categories which is the reason why the aggregate number of content exceeds the total number of tweets. The quantitative results of CorEx topic classification are presented in Table 4 (all providers) and Table 5 (grouped by provider).

**Table 4:** Content of the complete dataset (18,051 tweets) translated into English

Category (corresponding content in bold type)	Associated codes	Number of content (%)
1. News *Example: “****3 defeats in a row conceded the #SGE last. To make matters worse, they have a poor record against #HerthaBSC: No goal in the last 3 duels.**** Will #SGEBSC score something today? The bets for the game:* http://po.st/jcv4E*”* *(bwin Sportwetten, 2019–12-06)*	**sporting events,** match results, team line-ups, competitions, athletes, social projects, explanations on gambling	7224 (33%)
2. Product advertising *“Here we go! Will #BVB take the lead before the break like in the first leg? 1.60 – Dortmund score the 1st goal of the 1st half #BVBSLA live betting* http://po.st/rEelqM*”* *(bwin Sportwetten, 2019–12-10)*	drawing of lottery numbers, **sports betting events,** other lotteries, **URLs to specific offers**	4466 (21%)
3. Additional information *Example:* *“****Around € 45 million in today’s #LOTTO6aus49 jackpot – closing time 7 p.m.*** http://lotto-bayern.de/losgehts* #LegalbeimOriginal #6aus49”* *(LOTTO Bayern, 2020–11-28)*	odds, **prize amount**, **time and date of the gamble,** winning numbers	3802 (18%)
4. Marketing *“Today we have something outstanding for you! ****Win an online meet and greet with FC Red Bull Salzburg!**** How can you win this? Comment on this post, tell us which player you would like to meet virtually. We wish you good luck with the Tipico sweets!”* *(Tipico, 2020–11-13)*	brand promotion, engagement, celebrity endorsement, company information, **raffles**	2333 (11%)
5. Results *Example: “****The #lotto numbers 6aus49 of 20.11.2019: 2,7,10,18,31,42 SZ: 0.**** Did you win the 1 million euros? Check here: *https://t.co/Q0VaZbnpxy*”* *(Lottoland.com, 2019–11-20)*	winning totals, successful betting slips, **winning numbers**	1637 (8%)
6. Responsible Gambling *Example:* *“On Friday there is € 10 million in the #Eurojackpot jackpot* *Chance of winning 1:95 million //*http://lotto-bw.de***From 18. Addictive. Help at*** http://bzga.de*”* *(Lotto BW, 2019–11-25)*	responsible gambling and **harm-reductive content, age restrictions** (often pictured as symbols)	1179 (5%)
7. Other *Example: ****“@Weedogonzales***^***a***^*** Check with our support in live chat*** " *(Tipico, 2020–05-13)*	**provider response to user enquiries**	769 (4%)
8. Interaction *Example: “#RBLATL is the duel of last year’s finalists! Who will prevail?* ***Leipzig 66,7%*** ***Atletico 33,3%*** 9. votes – Final results” *(bwin Sportwetten, 2020–08-13)*	asking for feedback, **polls**, votes	230 (1%)

The analysis is based on 18,051 selected tweets during the observation period from 2019–03–27 to 2021–04–08. The number of content categories is higher than the number of tweets, as a tweet can be classified in several categories. The examples were translated by the authors

^a^ Weedogonzales is the name of a user who had asked for help

**Table 5:** Content categories by provider

Gambling provider	Number of tweets	Number of content categories	News (%)	Product advertising (%)	Additional information (%)	Marketing (%)	Results (%)	Responsible Gambling (%)	Other (%)	Interaction (%)	Coherence
*State lotteries*											
Lotto BW	2368	3150	5	18	27	11	8	25	5	−	0.44
LOTTO Bayern	1451	1801	3	34	36	1	22	−	4	−	0.48
*Social lotteries*											
Aktion Mensch e.V	3191	3244	81	−	−	15	−	−	5	−	0.45
Sportlotterie	66	76	51	14	3	32	−	− ^a^	−	−	0.56
*Lottery brokers*											
Lottoland.com	800	899	−	−	−	26	58	−	2	14	0.35
LOTTO24.de	468	851	4	20	27	5	12	23	9	−	0.57
*Casino*											
Spielbanken Bayern	285	420	10	−	12	55	14	9	−	−	0.49
*Sports betting*											
ADMIRALBET	2173	2717	29	39	21	11	−	−	−	−	0.45
bet-at-home	1186	1608	65	11	−	11	−	− ^a^	13	−	0.61
bwin Sportwetten	3197	3579	17	46	30	7	−	−	−	− ^a^	0.53
mybet	433	616	59	−	8	16	12	5	−	−	0.60
Tipico	597	608	15	15	14	20	13	11	12	−	0.50
Unibet Sportwetten	1836	2071	66	6	11	2	7	2	−	5	0.38

The analysis is based on 18,051 tweets during the observation period from 2019–03–27 to 2021–04–08. The number of content categories is higher than the number of tweets, as a tweet can be classified in several categories

^a^ This content is used by the providers, but cannot be captured by topic modeling. Even if there is no capture of the data, the categories are considered to be present in the evaluation

#### News

*News* was the most prominent category containing 33% of the overall content (Table 4). The *news*-category appears to be informative rather than promotional. Typically, the providers address sector-specific issues, e.g., information on sports teams and line-ups. Most Twitter messages from *Aktion Mensch e.V.* and the three sports betting providers *Unibet Sportwetten*, *bet-at-home* and *mybet*, contained *news* (81% and 66%, 65%, 59%, respectively), but only few from the state lotteries *Lotto BW* (5%) and *LOTTO Bayern* (3%) and none from the lottery broker *Lottoland.com* (Table 5).

#### Product Advertising

*Product advertising* accounts for 21% of the overall content. This category includes advertising for specific gambling products, e.g., a URL that directly leads to a certain betting offer. 46% of *bwin Sportwetten*’s and 39% of *ADMIRALBET*’s content fall under this category. In contrast, *Aktion Mensch e.V.*, *Lottoland.com*, *Spielbanken Bayern* and *mybet* did not use this category at all. Although *mybet as well as bwin Sportwetten and ADMIRALBET* offer the same range of products, they used *product advertising* to varying degrees.

#### Additional Information

Next in frequency was the category *additional information* (18%). This category includes supplementary information on gambling offers (e.g., odds or maximum winnings). Although some providers made use of this category (*LOTTO Bayern,* 36%, and *bwin Sportwetten,* 30%), it did not play any role for others (*Aktion Mensch e.V.*, *Lottoland.com* and *bet-at-home*). Again, no discernible trend evolved between providers of the same category.

#### Marketing

The percentage of overall content classified as *marketing* amounts to 11%. *Marketing* implies that providers promote their brand or advertise an engagement (e.g., team sponsoring); also, celebrity endorsement, raffles or company information belong to this category. Whereas the casino *Spielbanken Bayern* (55%) mostly relied on this category in its tweets, it played a minor role for some sports betting providers (e.g., *Unibet Sportwetten,* 2%), the lottery broker *LOTTO24.de* (5%) and the state lottery LOTTO Bayern (1%). Nonetheless, a clear trend between providers of the same sector could not be found.

#### Results

We classify 8% of the overall content as *results*. With the exception of *Lottoland.com* (58%), providers used *results*, such as winning totals or winning numbers, only rarely (which half of the sports betting providers did) or not at all (sports betting providers, social lotteries).

#### Responsible Gambling

Only 5% of the overall content classifies as *Responsible Gambling*. This category comprises harm-reduction and youth protection measures, for example, age limits. None of the tweets contained exclusively *Responsible Gambling* content and typically, the *Responsible Gambling* content was limited to an emoticon-sized symbol indicating the age limit.

*Responsible Gambling* content comprises 25% of *Lotto BW*’s and 23% of *LOTTO24*’s content, not in a very prominent form, as all tweets or images were merely supplemented by a subline referring to the age limit and potentially addictive character of the product. *Lotto BW* also adds the free hotline of the Federal Centre for Health Education to its tweets. Nevertheless, these providers did make use of this category, whereas five^10^ providers did not publish any harm-reductive content at all (*ADMIRALBET, Aktion Mensch e.V., bwin Sportwetten, LOTTO Bayern, Lottoland.com*), irrespective of the potential hazard of their products.

#### Other

The classification *other* accounts for 4% of the overall content. This category includes content that cannot be assigned to any other category, for example, responses to user enquiries. This category was rarely used except for *bet-at-home* and *Tipico*: 13% and 12%, of their tweets fell within this category, respectively.

#### Interaction

The classification *interaction* accounts for 1% of the content. This category refers to content that encourages users to interact either with the provider or the tweet, for example, requests for feedback, polls and votes. Only three providers worked with *interaction* in their tweets,^11^ and only one of them did so more intensely (*Lottoland.com*, 14%).

In all, the exploratory mixed-methods approach adopted was demonstrated to be appropriate for using the results of the qualitative content analysis to guide the quantitative semi-supervised Anchored CorEx model. For the 650 randomly selected tweets, the results of the summative content analysis and the CorEx model showed high coherence in 75% of all cases (Table 3). The coherence ranged from 0.45 to 0.63 (Table 13). Finally, the analysis of the complete dataset of 18,051 tweets shows high correspondence with the random sample, especially in the categories *news*, *additional information*, *results*, *Responsible Gaming*, *other* and *interaction* (Table 6).

**Table 6:** Relative number of content categories

Category	Random sample of tweets (*n*^a^ = 650)		Complete dataset (*n*^b^ = 18,051)
	Qualitative analysis (%)	CorEx (%)	CorEx (%)
1. News	31	16	33
2. Product advertising	15	21	21
3. Additional information	14	20	18
4. Marketing	17	19	11
5. Results	8	11	8
6. Responsible Gambling	9	7	5
7. Other	4	5	4
8. Interaction	1	2	1

^a,^^b^ *n* means the number of analysed tweets of the observation period from 2019–03–27 to 2021–04–08

## Discussion

In Germany, Twitter does not seem to be as important for providers of gambling products regarding scope, frequency of tweets and interaction as, for example, in the UK. In the present study, only one account has as many as 78,105 followers, whereas all others attract low four-figure numbers of followers or even less. In contrast, British sports betting providers may easily attract more than 100,000 followers (Bradley & James, 2019; Houghton et al., 2019; Killick & Griffiths, 2020). Possibly other social media are more important for providers operating in Germany. *Unibet Sportwetten*, for example, has only 4,500 Twitter followers, compared to 15,000 Instagram subscribers and 930,000 Facebook followers. Similar figures apply for the sports betting provider *bwin Sportwetten* and the state lotteries *Lotto BW and LOTTO Bayern*.

Similarly, all Twitter activities are rather low-level in our study. The providers send between 0.09 and 4.30 tweets per day, whereas Bradley and James (2019) report 89 tweets per day for the least active provider in the UK, Houghton et al. (2019) 25 and Killick and Griffiths (2020) report 33 tweets per day. Likewise, the most active provider in our study received 1.66 retweets and 4.66 likes per tweet, compared to 18.2 retweets and 72.8 likes by the sports betting provider *Paddy Power* in the UK (Bradley & James, 2019). However, the exact number of tweets that followers receive as push messages or that are displayed on their start screen also depends on the mechanics of Twitter’s timeline algorithm.

The potential of Twitter as an advertising, marketing and interaction channel has not yet been fully exploited by gambling providers in Germany, suggesting that there is a need for implementing the corresponding regulatory measures before advertising proliferates. Higher advertising volumes are to be expected with the admission of new forms of gambling into the German market by the new State Treaty of Gambling. Since exposure to gambling advertising might be positively associated with problem gambling (Syvertsen et al., 2021), an increase in the latter might be expected. This will fuel the demand for the regulation of gambling advertising that, thus far, has not been considered by regulators.

As both, the qualitative and quantitative analysis show, most of the content can be classified as *news*. The neutral presentation gives the tweets a professional and matter-of-fact touch, adding credibility to the provider. Despite minor differences between the categories *product advertising*, *additional information* and *results*, it is also evident that most tweets combine the *news* category with another, usually less neutral category. In our study, about one-fifth (21%) of the analysed content was direct advertising for gambling products, and a large share of the remaining tweets were directly linked to gambling products and gambling opportunities. Previous studies have shown that easy and fast accessibility, in combination with a permanent presence of gambling, contribute to the normalization of gambling, i.e., it becomes part of everyday life (Binde, 2007; Gainsbury et al., 2016a, b; McMullan & Miller, 2010; Sproston et al., 2015). The mere perception of gambling advertising can act as a trigger to participate in gambling, in particular, for disordered gamblers or persons who want to reduce or quit gambling (Binde, 2009; Hing et al., 2013, 2014). If information on and reminders about gambling become a daily companion for Twitter users, this is likely to contribute to more harm and vulnerability (Binde, 2007). Young people are at special risk, since children and youth have an increased risk for developing gambling problems (Derevensky & Gilbeau, 2015; Guillou-Landreat et al., 2021; Hanss et al., 2015; Li et al., 2018). This age group forms a large part of Twitter users. Moreover, our study has confirmed results from previous research that harm reductive or responsible gambling content rarely occurs (Bradley & James, 2019; Gainsbury et al., 2016b; Houghton et al., 2019; Killick & Griffiths, 2020; Sproston et al., 2015; Thomas et al., 2015; Torrance et al., 2021) and, even if it is present, it does not stand out.

With one exception, a clear pattern between the use of certain categories and belonging to a certain sector did not evolve in our study. The category *news* was preferentially used by sports betting providers as well as social lotteries and to a far lesser degree by the state lotteries, the lottery brokers and the casino. Krawczyk and Własiuk (2021) report more aggressive advertising slogans from providers of potentially less harmful products, such as lotteries, like *Aktion Mensch e.V.* and *Sportlotterie* in our case.^12^ So possibly, this category is used by providers of potentially more harmful products, like sports betting, to increase credibility and encourage the so-called “gamblification” of sports (Lopez-Gonzalez & Griffiths, 2018). However, it is equally conceivable that *news* relating to sports events are more attractive to a larger audience than *news* about lottery drawings. The use of all other categories was distributed unequally. The providers in our study may pursue different aims irrespective of the sector they belong to.

## Limitations and Future Directions

Although the observation period in our study covered more than two years, the results are not necessarily representative for longer and especially future periods. Providers might change their marketing strategies at short notice and/or flexibly adapt their appearance in social media. Most particularly, in the wake of the COVID-19 pandemic, the gambling market was subject to severe restrictions, especially in 2020. During the lockdown, land-based gambling services had to close and sporting events were temporarily banned (Auer et al., 2020; Håkansson et al., 2021; Nosal & Lopez-Gonzalez, 2021). It seems likely that the providers adapted their marketing strategies to the prevailing conditions.

Another limitation is of a technical nature. The Twitter API can only collect 3200 tweets per account. Some providers, whose accounts comprised more than 3200 tweets, had slightly lower numbers. The most likely explanation was that some tweets had been deleted by the providers themselves before the time of the investigation.

For topic modeling, the images used in the tweets are to some extent problematic. These are often accompanied by text, which is often only reproduced fragmentarily or incorrectly and thus cannot be used for the analysis. For this reason, the *Responsible Gambling* category was not taken into account for the providers *Sportlotterie* and *bet-at-home*. For example, in the second case, this was limited to an emoticon-sized symbol indicating the age limit.

A similar problem occurred with the provider *bwin Sportwetten*. This provider used polls to interact with its users. However, this Twitter feature could not be read out by the API, which is why the *interaction* category for this provider is not taken into account in the analysis.

Another challenging aspect of a semi-supervised approach can be the semantic spectrum of the related categories. It may prove difficult to describe these to the full extent and to attach specific anchor words to them. For example, the providers of sports betting report on different sports, different competitions and athletes in the tweets classified as *news*. They do not restrict their coverage to one country, i.e. one tweet is about football in Germany, the next about tennis in France. In any case, comprehensive preparatory work is required to determine the categories precisely in order to guide the algorithm to an accurate classification and to classify the remaining content into an open category *other*.

In general, we do not know how many users actually read a tweet and even if they do, if they are influenced by the tweets and to what degree. The sheer volume of the providers’ activities only permits limited conclusions on the effects on (potential) users. This could only be investigated in studies with users. Neither do we know who the users are—recreational or disordered gamblers, adults or minors. In general, social media appeal to young people. According to Twitter’s terms and conditions, an account can be created from the age of 13, but this might not prevent younger users from doing so. Participation in gambling however requires a minimum age of 18 in Germany. Therefore, future studies should assess the effects of tweets on the gambling attitudes, gambling intentions and gambling behavior of different user groups.

## Conclusion

Our paper is dedicated to filling a literature gap on the categorization of Twitter messages of the major German gambling providers using a novel approach, which combines qualitative and quantitative analysis. We used qualitative analysis performed by experts in the gambling research field in order to derive a categorization of topics and the related keywords. Afterwards we used these qualitative results as seeds, or namely guides, for the semi-supervised topic modeling using the CorEx model. The given mixed-methods approach revealed eight distinct topics, ranked according to their share in the complete corpus of Tweets: *news*, *product advertising*, *additional information*, *marketing*, *results*, *Responsible Gambling*, *other* and *interaction*. Our findings suggest that the agreement between the qualitative and the quantitative analysis with respect to these categories was relatively high.

The top category *news* can be considered as an indirect promotion of gambling services since information about sport events is communicated together with logos and colour schemes of the providers, inviting the customers to engage in gambling. The category *product advertising* relates to direct promotion and was on the second place for the complete corpus. Thus our findings indicate that indirect and direct promotion dominate in the corpus of the German gambling provider Tweet messages. The category *Responsible Gambling* has a share of only 5% for the corpus of Tweets with *interaction* having the lowest share. Therefore, we would like to highlight the importance of regulation of advertisements on social networks and media, since providers actively use platforms like Twitter for direct and indirect advertising. The fact that many persons from vulnerable target groups and in particular minors are actively using these social media platforms amplifies the policy implications of our findings.

The social media activities of gambling providers in Germany need to be understood in the context of the new State Treaty on Gambling in Germany. It is to be expected that the legalization of various forms of online gambling will lead to an increase in the social media activities of the relevant providers. Moreover, the State Treaty provides for liberal advertisement practices due to its generally worded regulations. Accordingly, the State Treaty (GlüStV, 2021) stipulates that advertising may not be *excessive*. Furthermore, minors and comparably vulnerable target groups may not be addressed explicitly, whereas minors are to be excluded as recipients of *advertising if possible*. Which advertising measures are considered *excessive*, and *whether it is possible* to exclude minors from advertising, not only involves numerous complex technical and legal aspects but is certainly viewed differently by stakeholders (gamblers, providers, addiction experts, regulators, policy makers). Most probably, these issues will become a matter for the German courts in the event of dispute. In this case, a considerable amount of time will pass before potential judicial clarification.

Thus, if such liberal handling leads to the population being massively penetrated by gambling advertisement, restrictions (including advertising bans), as in other countries [e.g., Australia, Belgium or Italy (Newall et al., 2019)], could be among potential consequences, for example in the form of binding advertising guidelines. Consequently, social media and Twitter in particular, which are not explicitly referred to in the German State Treaty on Gambling, could become significantly more important as advertising channels.

## Data Availability

The data compiled via Twitter API and analysed during the current study are available from the corresponding author on reasonable request.

## Appendix

See Tables 7, 8, 9, 10, 11, 12, 13.

**Table 7:** Frequency of tweets by provider

Gambling provider	Number of tweets	
	Total	Per day
*State lotteries*		
Lotto BW	2368	3.19
LOTTO Bayern	1451	1.95
*Social lotteries*		
Aktion Mensch e.V	3,191	4.29
Sportlotterie	66	0.09
*Lottery brokers*		
Lottoland.com	800	1.08
LOTTO24.de	468	0.63
*Casino*		
Spielbanken Bayern	285	0.38
*Sports betting*		
ADMIRALBET	2173	2.92
bet-at-home	1186	1.60
bwin Sportwetten	3197	4.30
mybet	433	0.58
Tipico	597	0.80
Unibet Sportwetten	1836	2.47

The analysis is based on 18,051 tweets during the observation period from 2019–03–27 to 2021–04–08

**Table 8:** Interaction with the tweets by provider

Gambling provider	Number of tweets			Retweets		Likes	
	Total	Per day	With own content	Total	Per tweet	Total	Per tweet
*State lotteries*							
Lotto BW	2368	3.19	2236	122	0.05	2272	1.02
LOTTO Bayern	1451	1.95	1429	514	0.36	2024	1.42
*Social lotteries*							
Aktion Mensch e.V	3191	4.29	3,166	5,271	1.66	14,738	4.66
Sportlotterie	66	0.09	66	20	0.3	53	0.8
*Lottery brokers*							
Lottoland.com	800	1.08	799	750	0.94	1592	2
LOTTO24.de	468	0.63	468	34	0.07	627	1.34
*Casino*							
Spielbanken Bayern	285	0.38	285	28	0.1	72	0.25
*Sports betting*							
ADMIRALBET	2173	2.92	2068	41	0.02	241	0.12
bet-at-home	1186	1.6	1182	74	0.06	900	0.76
bwin Sportwetten	3197	4.3	3175	240	0.09	1065	0.34
mybet	433	0.58	300	6	0.02	55	0.18
Tipico	597	0.8	423	67	0.16	1279	3.02
Unibet Sportwetten	1836	2.47	1760	594	0.33	5172	2.94

The analysis is based on 18,051 tweets during the observation period from 2019–03–27 to 2021–04–08

**Table 9:** Tweet content by provider

Gambling provider	Number of tweets					Replies	
	Total	Use images		Use URL			
		Total	(%)	Total	(%)	Total	(%)
*State lotteries*							
Lotto BW	2368	853	36.02	2457	82.84	178	7.52
LOTTO Bayern	1451	372	25.64	1051	72.38	62	4.27
*Social lotteries*							
Aktion Mensch e.V	3,191	597	18.69	3,015	91.92	147	4.61
Sportlotterie	66	30	45.45	62	91.18	3	4.55
*Lottery brokers*							
Lottoland.com	800	211	26.38	531	66.38	120	15.00
LOTTO24.de	468	236	50.43	160	33.97	189	40.38
*Casino*							
Spielbanken Bayern	285	268	94.04	183	61.41	3	1.05
*Sports betting*							
ADMIRALBET	2173	87	4.00	2538	92.53	12	0.55
bet-at-home	1186	550	46.37	493	41.39	168	14.17
bwin Sportwetten	3197	246	7.69	2207	68.97	172	5.38
mybet	433	253	58.43	181	41.51	15	3.46
Tipico	597	322	53.94	233	38.70	95	15.91
Unibet Sportwetten	1836	1718	93.57	815	43.96	29	1.60

The analysis is based on 18,051 tweets during the observation period from 2019–03–27 to 2021–04–08. When the number of URLs exceeds the number of tweets, several URLs were used in one tweet

**Table 10:** Top 10 hashtags of all providers

Hashtags	Number
lotto6aus49	1396
bundesliga	1250
jackpot	1227
unibet	1160
admiral	1104
eurojackpot	964
lottobw	764
inklusion	548
quotenboost	535
ucl	533

The analysis is based on 18,051 tweets during the observation period from 2019–03–27 to 2021–04–08

**Table 11:** Top 5 hashtags per provider

Gambling provider	Hashtag	Gambling provider	Hashtag
Lotto BW	lotto6aus49	LOTTO Bayern	jackpot
	lottobw		lotto6aus49
	jackpot		legalbeimoriginal
	eurojackpot		eurojackpot
	lottozahlen		6aus49
Aktion Mensch e.V	inklusion	Sportlotterie	sportlotterie
	behinderung		
	barrierefreiheit		
	corona		
	teilhabe		
Lottoland.com	euromillions	LOTTO24.de	lotto24
	lottozahlen		glücklichmacher
	eurojackpot		eurojackpot
	powerball		lotto
	lotto		lotto6aus49
Spielbanken Bayern	spielbankenbayern	bet-at-home	Blog
	spielbank		bundesliga
	casino		premierleague
	spielbanken		Ucl
	bayern		fcbayern
ADMIRALBET	admiral	mybet	fussball
	quotenboost		sport
	topquoten		sportwetten
	bonus		mybetmeister
	bundesliga		bundesliga
bwin Sportwetten	bundesliga	Unibet Sportwetten	unibet
	ucl		bundesliga
	bvb		bvb
	fcbayern		fcb
	b04fcb		Borussia
Tipico	bundesliga		
	topfakt		
	scheinderwoche		
	tipicotopfakt		
	zahlendesspieltags		

The analysis is based on 18,051 tweets during the observation period from 2019–03–27 to 2021–04–08

**Table 12:** Inter-rater reliability between summative content analysis and semi-supervised topic modeling

Gambling provider	Category	Fleiss’ κ	*p*-value
Lotto BW	K1	0.650	0.000
	K2	0.554	0.000
	K3	0.543	0.000
	K4	0.840	0.000
	K6	0.767	0.000
	K7	0.789	0.000
	K8	0.656	0.000
LOTTO Bayern	K1	0.917	0.000
	K2	0.876	0.000
	K3	0.781	0.000
	K4	0.789	0.000
	K7	1	0.000
	K8	0.656	0.000
Lottoland.com	K3	1.000	0.000
	K4	0.890	0.000
	K5	0.787	0.000
	K8	1.000	0.000
LOTTO24.de	K1	0.579	0.000
	K2	0.692	0.000
	K3	0.621	0.000
	K4	0.479	0.001
	K6	0.672	0.000
	K7	0.504	0.000
	K8	0.077	0.586
Aktion Mensch e.V	K4	0.096	0.498
	K7	− 0.042	0.768
	K8	0.645	0.000
Spielbanken Bayern	K2	1	0.000
	K3	0.458	0.001
	K4	− 0.389	0.006
	K6	0.645	0.000
	K7	0.608	0.000
Sportlotterie	K1	0.840	0.000
	K2	0.728	0.000
	K4	0.201	0.155
	K7	0.080	0.573
ADMIRALBET	K1	0.673	0.000
	K2	0.563	0.000
	K4	0.452	0.001
	K7	− 0.282	0.046
Bet-at-home	K1	0.543	0.000
	K4	0.912	0.000
	K7	− 0.250	0.077
	K8	0.240	0.149
Bwin Sportwetten	K1	0.756	0.000
	K2	0.578	0.000
	K4	0.368	0.009
	K7	− 0.639	0.000
Mybet	K2	0.285	0.044
	K3	0.185	0.191
	K4	0.766	0.000
	K6	0.534	0.000
	K7	0.015	0.917
Tipico	K1	0.357	0.012
	K2	0.003	0.981
	K3	0.811	0.000
	K4	0.299	0.035
	K6	0.534	0.000
	K7	− 0.515	0.000
	K8	− 0.136	0.335
Unibet Sportwetten	K1	0.905	0.000
	K2	− 0.033	0.813
	K3	1.000	0.000
	K4	0.539	0.000
	K5	0.558	0.000
	K6	0.368	0.009
	K7	− 0.683	0.000

**Table 13:** Content categories by provider (CorEx analysis of 50 tweets per provider)

Gambling provider	Number of content categories	Product advertising (%)	Additional information (%)	Results (%)	Marketing (%)	Interaction (%)	Responsible Gambling (%)	News (%)	Other (%)	Coherence
*State lotteries*										
Lotto BW	59	17	27	15	19	–	15	5	2	0.49
LOTTO Bayern	63	30	44	16	5	–	–	2	3	0.63
*Social lotteries*										
Aktion Mensch e.V	54	–	–	–	24	–	–	69	7	0.45
Sportlotterie	56	25	7	–	43	–	– ^a^	25	0	0.51
*Lottery brokers*										
Lottoland.com	56	–	–	64	20	14	–	–	2	0.61
LOTTO24.de	75	19	23	9	4	–	24	7	15	0.48
*Casino*										
Spielbanken Bayern	76	–	14	20	50	–	5	11	–	0.47
*Sports betting*										
ADMIRALBET	82	38	41	–	9	–	–	12	–	0.52
Bet-at-home	51	35	–	–	13	0	0 ^a^	31	20	0.50
bwin Sportwetten	56	48	25	–	5	–^a^	–	21	–	0.49
mybet	52	–	17	13	31	0	21	17	–	0.47
Tipico	61	31	15	8	13	0	16	5	11	0.46
Unibet Sportwetten	52	29	29	4	8	17	4	10	–	0.47

The analysis is based on 650 randomly selected tweets (50 tweets per provider) during the observation period from 2019–03–27 to 2021–04–08

The number of content categories is higher than the number of tweets, as a tweet can be classified in several categories

^a ^ This content is used by the providers, but cannot be captured by topic modeling. Even if there is no capture of the data, the categories are considered to be present in the evaluation

## Footnotes

1 *Unibet Sportwetten* offers sports betting, online slots and poker. On the company ‘s Twitter account, the sectors online slots and poker do not play a role during the observation period.

2 Of the two lottery brokers, *LOTTO24.de* holds a license for Germany, *Lottoland.com* does not.

3 During the work on the given paper the following version of Python had been used: 3.7.13.

4 In addition, several functions from packages Gensim (Rehurek and Sojka 2011), NLTK (Bird et al., 2009), NumPy (Harris et al., 2020) and Pandas (McKinney, 2010) were used.

5 For this purpose, the well-established “pytesseract PyPI” library available at https://pypi.org/ project/pytesseract/ has been applied. With only a few exceptions “pytesseract” successfully extracted short messages embedded into images (pytesseract, 2022).

6 In our context the lemmatizer demonstrated better results compared to conventional stemmers.

7 The only substantial deviation in κ was noted for the category *interaction*. Since 15 Twitter messages fall into this category, despite a high expected agreement rate of 90.72, minor mismatches between the two raters may explain the low κ value of 0.46. Another potential explanation could be the well-known paradox of the chance-correction ratio, which may result in a low κ value even under high agreement (see Feinstein and Cicchetti, 1990).

8 Theoretically, there would be 13 providers × 8 categories = max 104 categories to compare.

9 The decisions on the number of topics are based on the qualitative analysis. Although the coherence does not play a central role in our approach, we have decided to report it in order to show that the choice of topics is overall coherent with the corpus. The total correlation indicator, native to the CorEx method, was also relatively high in almost all cases, yet this indicator is not standardized and is not as intuitive as the coherence score (${C}_{v}$), which we use for the reporting purposes.

10 Although Table 5 shows that both providers *bet-at-home* and *Spielbanken Bayern* also do not use *Responsible Gambling* content, this is due to a technical limitation in the data collection. Consequently, the two providers are not mentioned here.

11 Due to a technical limitation in the data collection, the category *interaction* could not be considered for the provider *bwin Sportwetten*.

12 This could be due to the fact that potentially less harmful forms of gambling might have less strict advertising guidelines. The study by Krawczyk and Własiuk considers slogans from a wide variety of countries without investigating the differences between various countries and regulation systems, leaving this question unanswered.
